# Dorsal approach with tailored partial sacrectomy and gluteal V–Y fasciocutaneous advancement flap for the management of recurrent pelvic sepsis; case report

**DOI:** 10.1186/s12893-021-01189-0

**Published:** 2021-04-15

**Authors:** Sebastian Sparenberg, Sarah Sharabiany, Gijsbert D. Musters, Brenda M. Castano Borrero, Roel Hompes, Oren Lapid, Pieter J. Tanis

**Affiliations:** 1 Department of Surgery, Amsterdam UMC, University of Amsterdam, Meibergdreef 9, 1105 AZ Amsterdam, The Netherlands; 2 Department of Plastic, Reconstructive and Hand Surgery, Amsterdam UMC, University of Amsterdam, Meibergdreef 9, 1105 AZ Amsterdam, The Netherlands

**Keywords:** Dorsal approach, Pelvic sepsis, Sacrectomy, Flap reconstruction, Case report

## Abstract

**Background:**

Pelvic sepsis after surgery for rectal cancer is a severe complication, mostly originating from anastomotic leakage. Complex salvage surgery, during which an omentoplasty is often used for filling of the pelvic cavity, is seldomly required. If this fails, a symptomatic recurrent presacral abscess with a risk of progressive inflammation can develop. Such patients have often undergone multiple surgeries and have disturbed abdominal wall integrity, adhesion formation, and presence of one or two stoma(s). Subsequent salvage surgery via the conventional anterior abdominal approach is therefore less suitable. We describe three cases with a chronic presacral sinus and failure of first salvage surgery. All three patients underwent a prone only approach with tailored sacrectomy. This novel approach provided direct access to the pelvic abscess with optimal exposure for complete and safe debridement. A unilateral or bilateral gluteal V–Y fasciocutaneous advancement flap was created to completely fill the cavity with well vascularized tissue.

**Case presentations:**

Three male patients of 80, 66 and 51 years of age initially underwent low anterior resection with neo-adjuvant radiotherapy for rectal cancer. The first patients underwent intersphincteric resection of the anastomosis with omentoplasty 128 months after index surgery, and second salvage surgery 2 months later. The second patient underwent abdominoperineal resection with omentoplasty for locally recurrent rectal cancer, cystoprostatectomy with revision of the omentoplasty for pelvic sepsis 100 months after index surgery, and second salvage surgery 16 months later. In the third patient, the anastomosis was dismantled with subsequent intersphincteric proctectomy and omentoplasty 20 months after index surgery, and second salvage surgery was performed 93 months later. Second salvage surgery in all three patients was indicated because of symptomatic recurrent pelvic sepsis. Second salvage surgery consisted of sacrectomy, complete debridement of the presacral area, and filling with a gluteal advancement flap. This resulted in favorable postoperative recovery with ultimate healing of the pelvic cavity.

**Conclusion:**

The dorsal approach with tailored sacrectomy and gluteal V–Y advancement flap is a valuable option in highly selected patients to treat recurrent pelvic sepsis after multiple prior transabdominal interventions for chronic presacral sinus.

## Background

Pelvic sepsis is a dreaded complication after rectal cancer surgery. It might originate from anastomotic leakage or presacral abscess formation after abdominoperineal excision or low Hartmann’s procedure. Most of these problems resolve with conventional treatment strategies including dismantling of the anastomosis, endoscopic vacuum-assisted closure, a diverting stoma, and abscess drainage. But a chronic presacral sinus may develop in up to 9% of patients after rectal cancer surgery [1]. Patients with a history of previous radiotherapy are at particular risk for chronic pelvic sepsis [2].

In patients with symptomatic chronic pelvic sepsis and greatly affected quality of life, salvage surgery will be the backbone of the treatment. Bowel continuity can sometimes be preserved, but salvage surgery often includes intersphincteric resection of the leaking anastomosis or rectal stump with combined abdominoperineal debridement of the presacral sinus and fistula tracts, followed by filling of the pelvic cavity using an omentoplasty [3].

In some patients, salvage surgery fails with a residual or recurrent presacral abscess distal to the omentoplasty. This might originate from incomplete debridement with remaining (radiation) fibrosis, or an insufficient omentoplasty related to inadequate bulk or a relatively short pedicle. This can be an almost asymptomatic condition, but some patients get severe complaints and there is a risk of fistula formation. Those patients are difficult to manage, and surgical options are limited. One might consider further mobilization of the omentoplasty or a rectus abdominis muscle (RAM) flap, but this would again require an abdominal approach [4]. The anterior route might not be attractive due to disturbances of the abdominal wall, extensive intra-abdominal adhesions, scarring at the pelvic inlet, and the presence of a stoma.

In these challenging cases, we have developed the dorsal approach with a tailored partial sacrectomy in combination with a gluteal V–Y fasciocutaneous advancement flap. This case report describes three patients who underwent this novel procedure for recurrent pelvic sepsis following failed omentoplasty.

## Case presentation

Three patients who initially underwent neoadjuvant radiotherapy and low anterior resection (LAR) for rectal cancer underwent first salvage surgery for chronic presacral sinus between January 2011 and February 2020 at our academic hospital that is also considered a national referral centre for anastomotic failure. Patient characteristics are described in Table 1. The three patients were all male and between 51 and 80 years old at the time of second salvage surgery.

**Table 1:** Details of the surgical procedures with post-operative outcomes

	Patient 1	Patient 2	Patient 3
Age^1^	80	66	51
BMI	26.5	28.1	30.0
Gender	Male	Male	Male
Comorbidity	–	Hypertension, diabetes Myocardial infarction Occlusive peripheral arterial disease Prostate cancer	Pulmonary embolism
Pelvic radiotherapy	5 × 5 Gy	5 × 5 Gy Brachytherapy prostate	Chemoradiotherapy
Surgical History for underlying disease	LAR for ypT1N0M0 rectal cancer with temporary ileostomy Endoscopic dilation of anastomotic stenosis	LAR for ypT3N0M0 rectal cancer with temporary ileostomy Segmental small bowel resection for enterocutaneous fistula APR for locally recurrent rectal cancer with omentoplasty	Double loop colostomy for obstruction, LAR for ypT2N0M0 rectal cancer, dismantling anastomosis with end-colostomy for leakage
First salvage surgery			
Indication	Chronic presacral sinus, with purulent discharge, bleeding and anemia	Chronic pelvic abscess with involvement of prostate, perineal fistula to the bladder	Chronic presacral sinus with debilitating discharge
Time from index surgery	128 months	100 months after LAR 36 months after APR	20 months
Operative details	Intersphincteric resection anastomosis with omentoplasty and end colostomy	Cystoprostatectomy, urostomy using colon conduit, transverse end colostomy and revision omentoplasty	Intersphincteric resection rectal stump, omentoplasty, incisional hernia repair
Postoperative complications	Persistent presacral abscess, treated with surgical drainage/endosponge	Persistent pelvic abscess,	Ileus due to adhesions on omentoplasty, treated with ileocecal resection Persistent presacral abscess, drainage procedures
Partial sacrectomy and gluteal VY fasciocutaneous advancement flap			
Indication	Pelvic sepsis with fever, severe pain and purulent discharge	Pelvic abscess with debilitating purulent discharge	Recurrent presacral abscess after symptom free interval of 6 years, pain, fever
Time from first salvage surgery	2 months	16 months	93 months
Operative details	Sacrectomy S3 unilateral VY from left buttock	Sacrectomy S4 bilateral VY	Sacrectomy S4, revision omentoplasty unilateral VY from left buttock
Duration of surgery (h)	4:14	4:07	3:23
Hospital stay (days)	15	12	5
Vacuum drain removal (days after surgery)	13	14	17
Post-operative complications	Fluid collection, percutaneous drainage and prolonged antibiotics	Small perineal sinus, healed with conservative management	Persisting pain, slowly improving during 12 months
Follow-up	3 months	48 months	21 months
Pelviperineal status	Wound healed No signs of recurrent abscess, diminishing pain	Healed perineum with good quality of life until death of recurrent cancer	Healed perineum, no signs of recurrent abscess, good quality of life

^1^Age at time of partial sacrectomy and gluteal VY fasciocutaneous advancement flap; *BMI* body mass index

The first patient developed anastomotic leakage after LAR, and the diverting stoma was closed after assumed healing of the leak. He presented with rectal blood loss, a major low anterior resection syndrome ten years after index surgery, and a chronic pre-sacral sinus was diagnosed. The persistent nature of the pelvic sepsis, unresponsive to antibiotics and drainage procedures, led to tertiary referral for salvage surgery 128 months after the LAR. Initial salvage surgery with intersphincteric resection of the anastomosis and omentoplasty was unsuccessful, requiring second salvage surgery 3 months later.

The second patient had undergone a LAR for rectal cancer and developed an enterocutaneous fistula after reversal of the diverting ileostomy. Tertiary referral and fistula closure followed. Five years later, locally recurrent rectal cancer was diagnosed during preoperative screening for prostate cancer, for which he underwent APR with an omentoplasty. This procedure was complicated by a pre-sacral abscess with subsequent involvement of the prostate and urinary fistula, which was treated by radiological drainage and a urinary catheter. After 3 years of recurrent urinary tract infections and failed conservative treatment of the fistula, the patient underwent a cystoprostatectomy with revision of the omentoplasty. This first salvage surgery was complicated by a persistent pre-sacral abscess unresponsive to radiological drainage and antibiotics, and a second salvage surgery took place 16 month after the cystoprostatectomy.

In the third patient, the LAR was complicated by anastomotic leakage, which persisted despite endosponge treatment. The anastomosis was dismantled with construction of an end colostomy 8 months after the LAR. The patient was referred because of a persisting abscess on the rectal stump, for which he underwent first salvage surgery with intersphincteric proctectomy and omentoplasty 20 months after index surgery. Although this was complicated by a persisting presacral abscess, the patient eventually recovered and had an asymptomatic period for 6 years. However, symptomatic recurrent pelvic sepsis was diagnosed. Initially, the abscess was percutaneously drained. Because of the persisting abscess, second salvage surgery was performed 93 months after the first salvage surgery.

### *Second salvage surgery *via* dorsal approach*

**Fig. 1:**
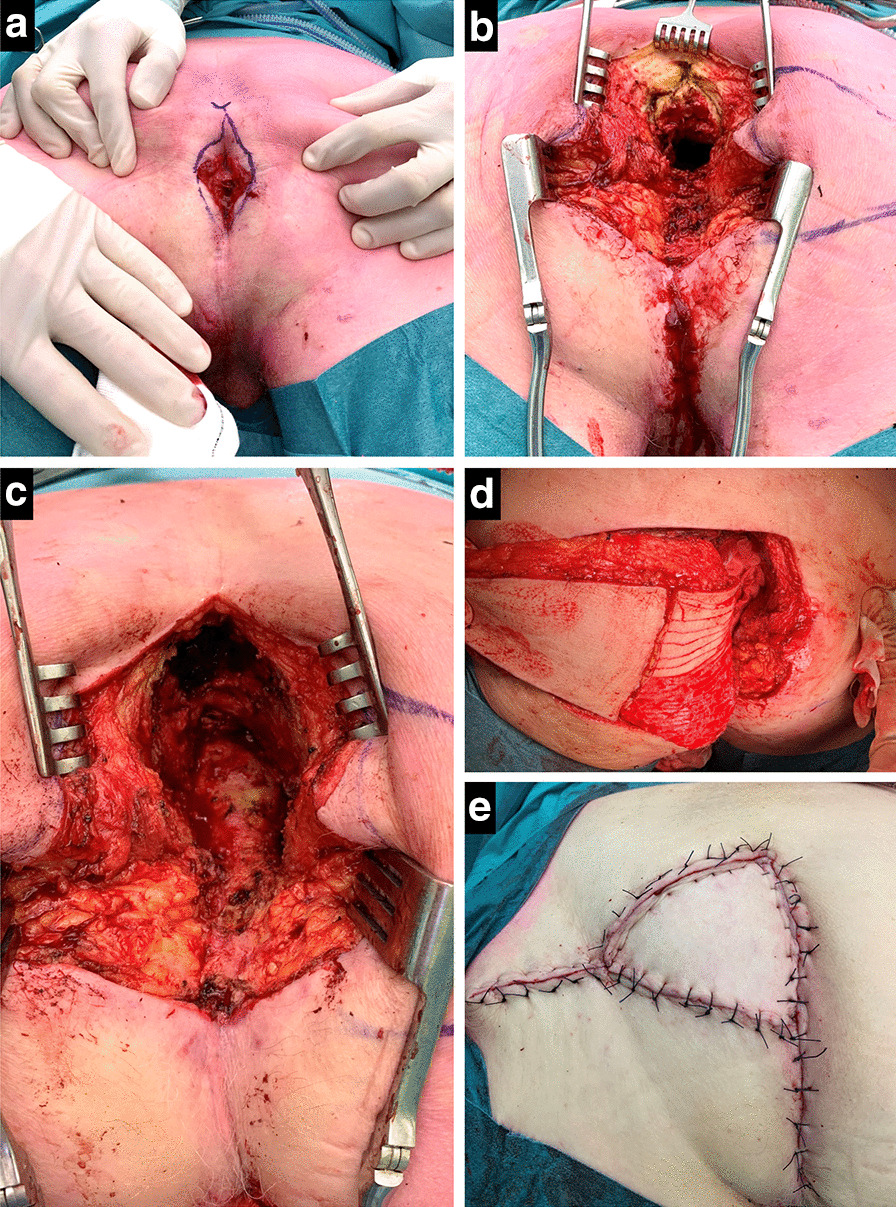
Second salvage surgery for recurrent pelvic sepsis in the first patient. The perineal fistula **a** was excised and sacrectomy at the level of S5 performed (**b**). Sacrectomy was extended with transection below S3 for optimal debridement (**c**). The gluteal V–Y advancement flap was created and the medial part deepithelialized (**d**), with layered closure (**e**)

Tailored sacrectomy with gluteal V–Y fasciocutaneous advancement flap was performed in all three patients, with optimal pelvic debridement and complete filling of the cavity. After general anesthesia and prophylactic antibiotics, the patient was positioned in prone position with the lower extremities in a split-leg table position. The previous perineal scar was excised, including any fistula tracts in the midline (Fig. 1a). The distal sacrum was exposed by dividing the lumbodorsal fascia and gluteal muscle attachments. The level of sacral transection required for adequate access was estimated based on preoperative imaging by drawing a horizontal line from the cranial edge of the abscess cavity on sagittal CT or MRI. Intraoperatively, sacrectomy was started at or below the level of S5 using an osteotome or oscillating saw. The sacrectomy was extended to a higher level if there was insufficient exposure or if more space was needed to bring the medial aspect of the V–Y flap into the cavity without any residual presacral dead space (Fig. 1b). After the distal sacrectomy, complete debridement of the abscess cavity follows, including excision of all radiation fibrosis until complete debridement without remaining sinuses or pockets is achieved (Fig. 1c).

**Fig. 2:**
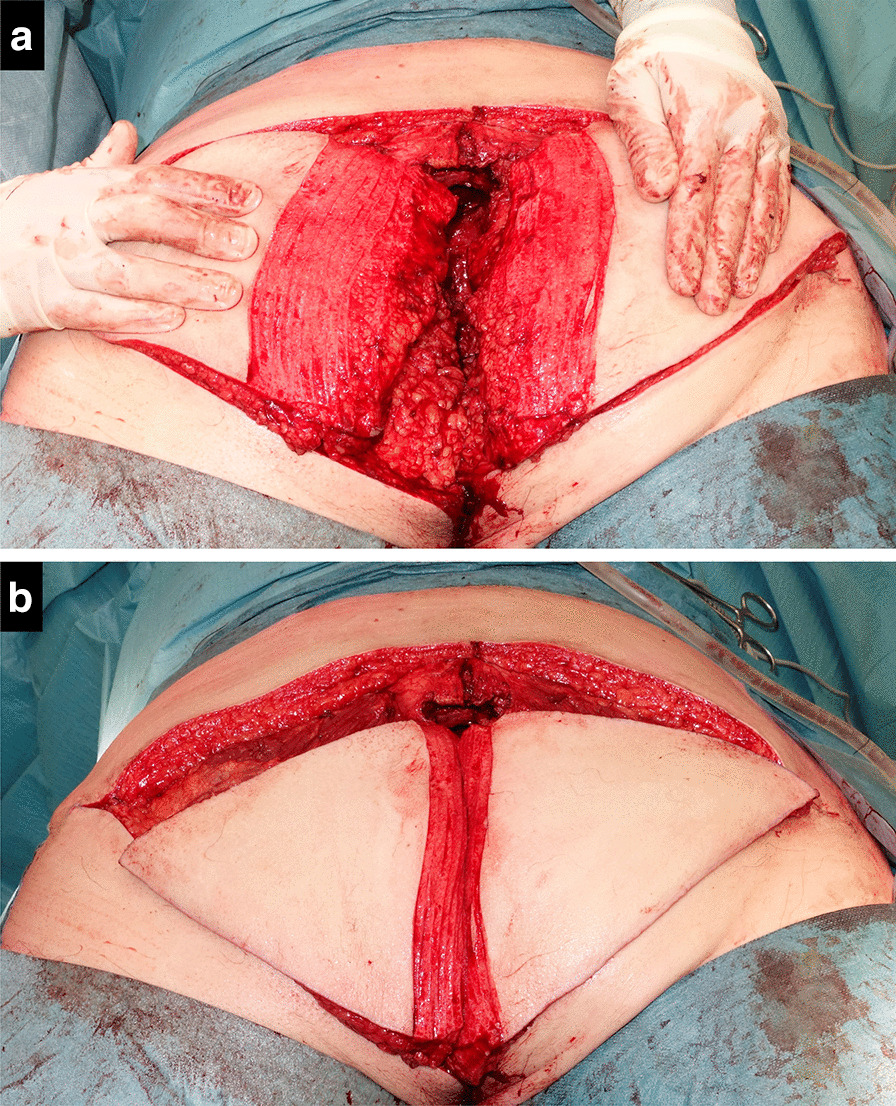
Second salvage surgery in which bilateral gluteal V–Y advancement flaps were created for adequate filling of the large pelvic cavity after sacrectomy below S4. The areas of deepitheliazed skin **a** illustrate the bulk of tissue that is being brought into the cavity (**b**)

**Fig. 3:**
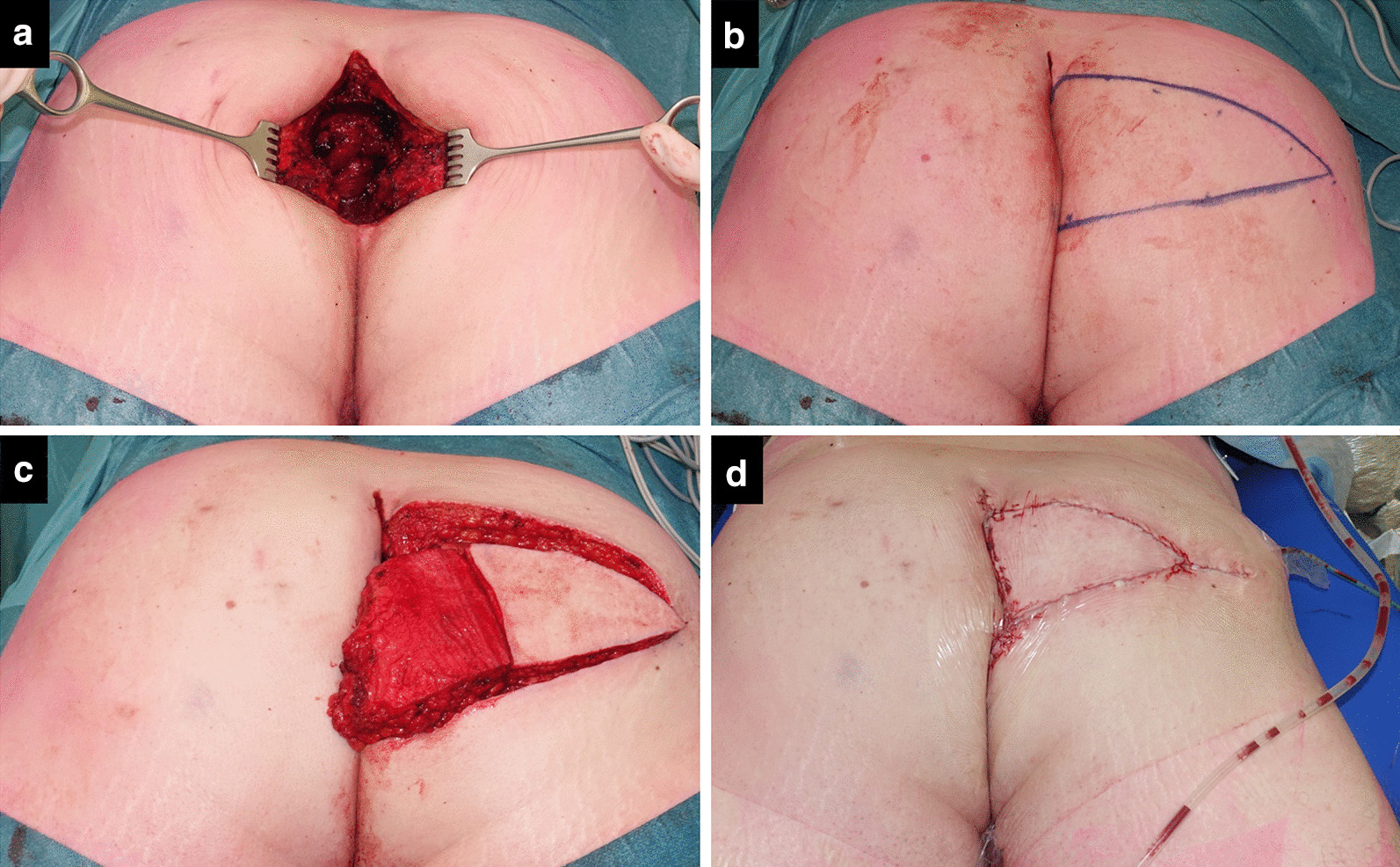
Second salvage surgery in the third patient, showing the confined but deep cavity (**a**), design of the flap (**b**), prepared flap with deepithelialized skin and a vertical back cut of a few centimeters (**c**), and postoperative status with vacuum drains positioned at the bottom of the cavity via perineal route and at the donor site (**d**)

The V–Y advancement flap was pre-marked on one of the buttocks, based on an estimation of the bulk and width required to fill the whole cavity. A V-shaped incision over the right or left gluteal region was performed, after which the subcutaneous fat and gluteal fascia was divided. The fasciocutaneous flap was partially dissected from the gluteal muscle until enough mobility was obtained to invert the flap into the cavity, carefully preserving the medial attachments containing the gluteal perforators. Only subcutaneous fat is used for obliteration of the cavity, without transposing the gluteus maximus muscle. The skin that would be turned inside was marked and de-epithelialized (Fig. 1d). In the second patient, the defect was insufficiently filled with the advanced flap, and a second flap from the contralateral site was created using the same technique (Fig. 2). In the third patient, a back cut in the cranial part of the deepithelialized flap was made to better conform the flap to the shape of the cavity (Fig. 3). After turning the flap into the cavity a vacuum drain was placed underneath the flap inside the cavity. Vicryl 2–0 sutures were used to fix the flap at the deepest point of the cavity. Afterwards, a layered closure of the subcutaneous fat was performed with Vicryl 2–0 and 3–0 while the skin was sutured either transcutaneously or intracutaneously (Fig. 1e). An additional vacuum drain was placed at the donor site when necessary.

To decrease pressure on the transposition flap, an anti-decubitus mattress was used. Patients were immobilized for no longer than 3 days, and were only allowed to stand or walk without flexion of the hip for the first week. The pelvic vacuum drain was kept in place for at least 1 week, and removed when the production was less than 10 cc per day.

### Postoperative outcome

**Fig. 4:**
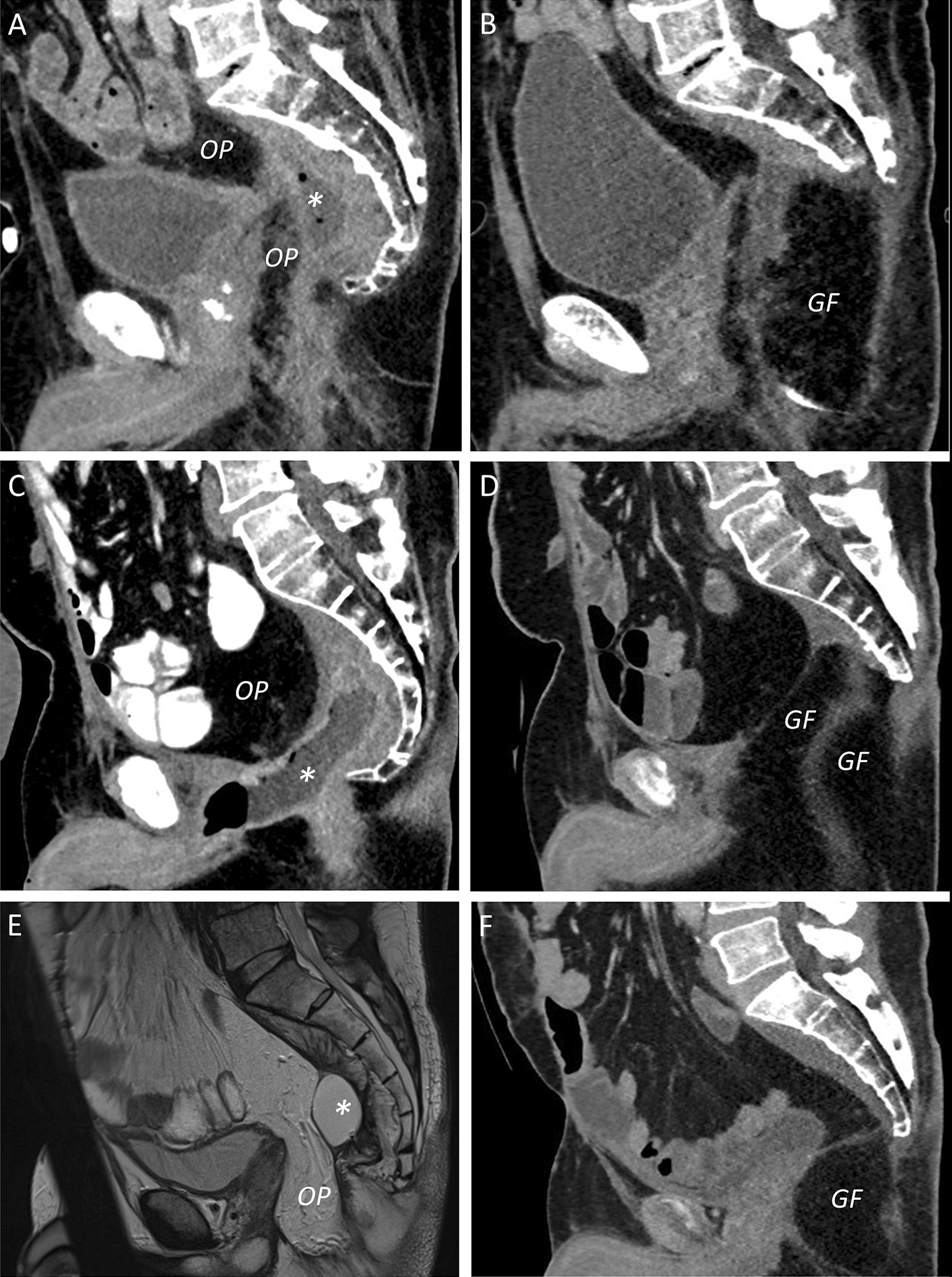
Pre- and postoperative sagittal pelvic imaging using CT (**a**–**d**, **f**) or MRI (**e**), showing the recurrent and persisting abscess (*) despite omentoplasty (OP) and drainage procedures, and subsequent postoperative result after distal sacrectomy with gluteal V–Y fasciocutaneous advancement flap (GF) in the first (**a**, **b**), second (**c**, **d**), and third patient (**e**, **f**). Some residual fluid below the flap was observed in the first patient (**b**), which was successfully drained

Following second salvage surgery, the first patient developed a fluid collection below the flap, for which successful percutaneous drainage was performed. The second patient had a small perineal sinus which spontaneously healed. The third patient had uncomplicated wound healing, but resolution of postoperative pain took one year. None of the patients had complaints suggesting sacral nerve injury and the perineal wound was healed with good quality of life until death from recurrent cancer (second patient) or at last follow-up (first and third patient). Figure 4 shows pre- and postoperative sagittal pelvic images of second salvage surgery.

## Discussion and conclusions

Chronic pelvic sepsis after rectal cancer surgery is a rare problem with high level of complexity that requires specialized care. Our unit became a national referral centre for anastomotic failure and refractory pelvic sepsis in the past decade. With increasing experience in salvage surgery, results improved over the years; however, incidentally we observed persisting or recurrent pelvic sepsis after major abdominoperineal salvage surgery with omentoplasty. The results of the three patients discussed reinforce our reluctance to choose an abdominal approach again. The dorsal approach with partial sacrectomy and gluteal V–Y fasciocutaneous advancement flap resulted in full control of pelvic sepsis in all three patients.

Partial sacrectomy has been described in patients with malignancies, but not for recurrent presacral abscess [5, 6]. Prone sacrectomy to avoid an abdominal approach has recently been described by Solomon et al. in two patients with dysplasia to perform completion proctectomy and pouch excision, respectively [7]. Although largely identical regarding the approach, our patients had the additional problem of a non-collapsing cavity with longstanding infection that needed tissue filling. As a consequence of the fibrotic walls of the cavity, there seems to be no risk of perineal herniation despite distal sacrectomy, based on our experience. The fact that Solomon et al. used a mesh to prevent perineal herniation suggests that the surrounding tissues in their patients were still compliant enough to fill the dead space after resection. We agree with Solomon et al. that the prone only approach with sacrectomy is feasible and safe, with excellent vision and access.

The presented salvage procedure uses a well-vascularized gluteal V–Y fasciocutaneous advancement flap that appears to have sufficient mobility to fill relatively deep pelvic cavities. A review of flap reconstructions after sacrectomy found that gluteal flaps were most frequently used, with the advantages of proximity to the defect and robust blood supply [8]. Gluteal flaps can either be advancement or rotation flaps, with or without muscle. Disadvantages that have been mentioned are related to limited reach of the flap and inadequate bulk, as well as compromised vascularisation after ligation of the internal iliac artery or gluteal arteries. Furthermore, concerns have been raised about prior pelvic radiotherapy and ambulatory function. With the donor site largely located outside the regular radiation fields for rectal cancer, we did not consider this a contraindication. Furthermore, if the muscle is not included in the flap, postoperative patients can be mobilized relatively early without long-term walking problems.

Alternative flaps described in the review of Asaad et al. include vertical rectus abdominus muscle flap (VRAM), anterolateral thigh and vastus lateralis flaps, free latissimus dorsi flap and some miscellaneous options for reconstruction [8]. The VRAM flap typically offers enough tissue for adequate filling, but compromises abdominal wall integrity and depends on preserved inferior epigastric vessels [2].

When facing chronic pelvic sepsis in patients that have had multiple previous surgeries, the anterior abdominal approach becomes more difficult and increases the risk for bowel injury or other injuries, for example to the bladder or ureter [9]. By entering the pelvic region dorsally after partial sacrectomy, optimal access to the septic pelvis is achieved, and the risk of intraoperative complications and surgical morbidity is decreased at the same time. It is also time efficient to perform pelvic debridement in the prone position, as illustrated by the relatively short duration of surgery, with an average of approximately 4 h (Table 1), in view of the underlying complexity of the problem. Routine CRP measurement and low-threshold for CT imaging post-operatively seem effective for early detection and drainage of fluid collections that develop despite placement of vacuum wound drains, as illustrated by patient 1. Wound healing was remarkably good in all three patients, which is in line with recent literature exemplifying the value of gluteal flaps after extensive pelvic surgery [10].

In conclusion, the dorsal approach with partial sacrectomy in combination with a gluteal V–Y fasciocutaneous advancement flap is a valuable option for a highly selected group of patients to treat recurrent pelvic sepsis after extensive previous transabdominal surgery. The well vascularized tissue transfer from the gluteal region achieves excellent filling of the pelvic space with favourable wound healing.

## Data Availability

Not applicable.

## Abbreviations

APR: Abdominoperineal resection
CT: Computed tomography
CRP: C-reactive protein
LAR: Low anterior resection
RAM: Rectus abdominis muscle
VRAM: Vertical rectus abdominis muscle
